# Extracellular Vesicle-Mediated Cell–Cell Communication in the Nervous System: Focus on Neurological Diseases

**DOI:** 10.3390/ijms20020434

**Published:** 2019-01-20

**Authors:** Celeste Caruso Bavisotto, Federica Scalia, Antonella Marino Gammazza, Daniela Carlisi, Fabio Bucchieri, Everly Conway de Macario, Alberto J. L. Macario, Francesco Cappello, Claudia Campanella

**Affiliations:** 1 Department of Biomedicine, Neuroscience and Advanced Diagnostics (BIND), Section of Human Anatomy, University of Palermo, 90127 Palermo, Italy; celestebavisotto@gmail.com (C.C.B.); scalia.fede@gmail.com (F.S.); antonella.marino@hotmail.it (A.M.G.); fabiobuk@gmail.com (F.B.); claudiettacam@hotmail.com (C.C.); 2 Euro-Mediterranean Institute of Science and Technology (IEMEST), 90136 Palermo, Italy; ajlmacario@som.umaryland.edu; 3 Institute of Biophysics, National Research Council, 90143 Palermo, Italy; 4 Department of Biomedicine, Neuroscience and Advanced Diagnostics (BIND), Section of Biochemistry, University of Palermo, 90127 Palermo, Italy; daniela.carlisi@unipa.it; 5 Department of Microbiology and Immunology, School of Medicine, University of Maryland at Baltimore-Institute of Marine and Environmental Technology (IMET), Baltimore, MD 21202, USA; econwaydemacario@som.umaryland.edu

**Keywords:** exosomes, extracellular vesicles, nervous system, central nervous system, cell–cell interaction, biomarkers, theranostics tools, neurological diseases

## Abstract

Extracellular vesicles (EVs), including exosomes, are membranous particles released by cells into the extracellular space. They are involved in cell differentiation, tissue homeostasis, and organ remodelling in virtually all tissues, including the central nervous system (CNS). They are secreted by a range of cell types and via blood reaching other cells whose functioning they can modify because they transport and deliver active molecules, such as proteins of various types and functions, lipids, DNA, and miRNAs. Since they are relatively easy to isolate, exosomes can be characterized, and their composition elucidated and manipulated by bioengineering techniques. Consequently, exosomes appear as promising theranostics elements, applicable to accurately diagnosing pathological conditions, and assessing prognosis and response to treatment in a variety of disorders. Likewise, the characteristics and manageability of exosomes make them potential candidates for delivering selected molecules, e.g., therapeutic drugs, to specific target tissues. All these possible applications are pertinent to research in neurophysiology, as well as to the study of neurological disorders, including CNS tumors, and autoimmune and neurodegenerative diseases. In this brief review, we discuss what is known about the role and potential future applications of exosomes in the nervous system and its diseases, focusing on cell–cell communication in physiology and pathology.

## 1. Exosomes, Microvesicles for Cell–Cell Communication and Tissue Homeostasis

Eukaryotic cells in multicellular organisms need to communicate with each other in order to maintain tissue homeostasis and to respond to pathogens in the extracellular milieu. Generally, cells exchange information through direct cell–cell contact or by secretion of soluble factors [1]. Mechanisms of intercellular interaction are known that involve the production and release of extracellular vesicles (EVs). Cells interact and influence the extracellular environment and other cells in various ways, for instance by releasing different types of EVs, which serve various functions depending on their origin and molecular composition. EVs include a variety of nanoscale membranous vesicles that are released by many cell types into the extracellular environment and can reach virtually all parts of the body [2]. EVs carry molecules such as nucleic acids, proteins, and lipids to specific target cells and can be classified according to their size, biogenesis, functions, and composition [3,4]. There are three main types of EVs: (1) microvesicles (100–1000 nm in diameter); (2) apoptotic blebs (1000–5000 nm in diameter); and exosomes (diameter 20–150 nm). The former two represent heterogeneous populations of vesicles generated by outward budding of the plasma membrane. Exosomes instead are generated by invagination of endosomal membranes and subsequent production of multivesicular bodies (MVBs) [5,6]. Frequently, in the literature, the terms exosomes and EVs are used imprecisely, most likely because a standardized, uniformed method for their isolation–characterization is not used universally and, therefore, the results vary among laboratories. Nevertheless, because of the increasing interest in EVs and because exosomes are currently the best characterized among them, in this review we will focus on the latter.

It was initially thought that exosomes could be a mechanism for shedding the cytoplasm in maturing sheep reticulocytes [7]. Later, it was demonstrated that exosomes are active players in intercellular communication [8,9,10,11], originate in endosomes and are secreted by all cell types, including neurons, under physiological and pathological conditions [12]. Exosomes are present in body fluids such as blood; urine; breast milk; saliva; and cerebrospinal, bronchoalveolar lavage, ascitic, and amniotic fluids [11,13,14,15,16,17,18,19,20,21].

Exosomes are released into the extracellular space after the merging of late endosomes with the cell membrane. Previously, early endosomes become part of multivesicular bodies (MVBs), which undergo a maturation process characterized by a gradual change in protein composition of the vesicles (intraluminal vesicles, ILVs). During this maturation process, the vesicles that have accumulated in the MVBs can follow three different pathways: (1) merge with the lysosomes, which leads to the degradation of their protein cargo (e.g., in the case of signalling receptors); (2) constitute a temporary storage compartment; and (3) blend with the plasma membrane, releasing exosomes. MVBs merge with the plasma membrane, resulting in exocytosis of the vesicles contained in them so that the vesicles’ membrane maintains the same topological orientation as the plasma–cell membrane [1,22,23]. The endosomal sorting complexes required for the transport machinery (constituted of the proteins ESCRT-0, -I, -II, -III) is involved in exosome biogenesis and loading [24]. ESCRT-1 assists in the sorting of the ubiquitinated cargo proteins at the endosome membrane and the ESCRT-associated protein ALIX (apoptosis-linked gene 2-interacting protein X) can regulate this function [24,25].

The content of exosomes reflects that of the cell of origin and, consequently, there is interest in characterizing it to obtain information on the cell of origin and the functions of exosomes, and to assess the potential of exosomes as drug delivery tools. The composition of exosomes depends on parental cell conditions, and includes lipids; proteins; and nucleic acids, such as DNA, non-coding RNA, rRNA (ribosomal RNA) and miRNAs (microRNAs) [26].

The lipid composition of exosomes is characteristic and includes cholesterol, phosphatidylcholine, sphingolipid ceramide, and sphingomyelin that probably stabilize the exosomal bilayer membrane and maintain its integrity in the extracellular milieu [27]. The sphingolipid ceramide plays a key role in the budding of exosomes [28].

Various classes of proteins are found in exosomes, such as proteins involved in the vesicles’ trafficking, cell surface receptors, and proteins involved in endocytic pathways (GTPases; annexins; flotillin; endosomal sorting complex required for transport, ESCRT, such as Alix; tumor susceptibility gene 101, TSG101; integrin; and a number of tetraspanins such as CD9, CD53, CD63, CD81, and CD82, depending on the cell of origin). Also, in exosomes are proteins with specific post-translational modifications (PTMs) [29,30], and proteins that are important in long-distance communication, such as cytokines [31], hormones [32], growth and transcription factors [33], and heat-shock proteins (HSPs) [10,30,34,35].

The presence of mRNA [36] and miRNA [37,38,39,40,41] in exosomes indicates activity in the regulation of gene expression in both recipient and donor cells, suggesting horizontal transfer of genetic information [42].

Depending on the parental cells and their contents, exosomes may have many different functions. They are involved in cell-to-cell information transfer [43], immune response [44], inflammation [45], coagulation [46], stem cell activation [47], and programmed cell death [48]. Exosomes can participate in cellular responses against stress [49]. It has been shown that exposing B-cell lines to heat stress results in a marked increase of HSPs in exosomes and in an increase in the quantity of exosomes produced [10,11,30,49,50,51].

Since exosomes can mediate transfer of molecules, it is very likely that they play a key role in intercellular interactions and in the maintenance of tissue homeostasis [52,53]. For example, exosomes play physiological roles in neuronal development, transmission of electrical impulse, and regeneration, and, consequently, can play a pathogenic role in neurological disease [54].

## 2. Nervous System Cells and Tissues: An Overview

The nervous system, composed by the central nervous system (CNS), and the peripheral nervous system (PNS), is implicated in the communication with both the external and internal environment of the organism by responding to chemical and physical stimuli [55].

**Figure 1:**
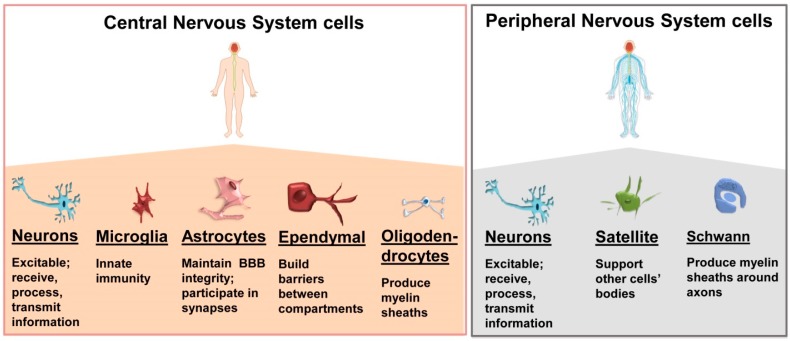
Cells of the central and peripheral nervous systems. These cells have functions and locales of residence distinctive of each of them but they all can secrete exosomes and receive exosomes from the others, as depicted in Figure 2. BBB: blood–brain barrier.

The main cell types found in the nervous tissue are neurons, or nerve cells, that have the ability to rapidly receive and transmit impulses to and from different parts of the body, and neuroglia, or glial cells, which assist in the propagation of the nerve impulses and provide nutrients to the neurons (Figure 1). Both neurons and neuroglia cells develop from the dorsal ectoderm of the early embryo but different types of them can be distinguished, which are characteristic of the CNS or PNS (Figure 1). Overall, these cells are responsible for most of the functional features of nervous tissue [56]. Since exosomes can be specific for the cell type that produce them, we briefly recapitulate the main features of the cells that constitute the nervous tissue and that, when their homeostasis is affected, can be implicated in the pathogenesis of nervous system diseases.

Neurons are highly specialized cells that receive, process, and transmit information through chemically-mediated electrical signals [56] (Figure 1).

Despite the fact that neurons can be specialized and differ in a variety of features, they all share several characteristics. The key function of neurons is to communicate between them and with other cell types. When the nerve impulses travel along the axon in the form of an action potential, the vesicles at the axon terminal, which contain neurotransmitters or neuromodulators, release their content by exocytosis. These signals between neurons are passed via specialized connections called synapses [57], in which either the axon terminal or an en passant bouton (a type of terminal located along the length of the axon) of one cell contacts another neuron’s dendrite, soma or less commonly, axon [57]. The chemical transmitters travel across the synaptic cleft to reach receptors on the postsynaptic cell. According to the neuron doctrine founded by Ramόn y Cajal and then supported by subsequent investigators, this seems an appropriate description for most synapses in vertebrates and invertebrates, but several studies and new technology applications, such as electron microscopy, have pointed out the existence within CNS of new synapses, called mix synapses and synapses à distance, where the axon and the dendrites appear to be exchanging their roles. It could lead to reform of the neuron primary doctrine and render it more pliable [58]. In the last decade, in vitro studies demonstrated that, depending on synaptic activity, neurons release exosomes that can be retaken by other neurons suggesting a novel way for inter-neuronal communication [59].

The term glia derives from the ancient Greek word “glía” meaning “glue” in English, and may suggest a passive type of cell; however, glial cells are active, providing support and nutrition to the neurons, form myelin, and, by insulating axons, speed up electrical communication [60]. A major distinction between glia and neurons is that glia do not participate directly in synaptic interactions and electrical signaling. However, emerging evidence suggests that glia, particularly astrocytes, are involved in the formation of synapses and in modulating synaptic function through bidirectional communication with neurons, both during development and in adulthood [60].

For many years it has been argued that the number of glial cells in the brain was significantly higher than neurons, but recent work has revealed that neurons and glia are almost equal in number in the human cortex [61,62,63]. However, it is possible that the proportions of neurons and glial cells vary in different brain areas [64].

Neuroglia of the CNS can be divided into macroglia and microglia (Figure 1). The macroglia includes oligodendrocytes, astrocytes, and ependymoglial cells that originate from the ectoderm, while the microglial cells derive from the yolk sac and they are found in the CNS during early embryonic development [65].

Spinal cord and brain contain different subclasses of oligodendrocytes (OLGs) which derive from multiple sources [66] (Figure 1). OLGs provide a lipid-based insulation and, thus, increase the speed at which the action potential can travel in the axon.

Within the oligodendrocyte linage, there exist the NG2-glia/oligodendrocyte cells. They are characterized by the presence on their surface of chondroitin sulfate proteoglycan and are considered an independent glial population, but their function in the adult brain is not yet fully established. NG2-glia maintains the physiological and homeostatic conditions of the nervous tissue generating mature myelinating oligodendrocytes; furthermore, it forms synapses with neurons of the hippocampus and probably in other parts of the brain, too [67]. Notably, NG2-glial cells have the ability to receive signals without creating or propagating action potentials [67].

The astrocytes are supportive glial cells in neural tissue with a star-like appearance because of their elaborate cytoplasmic processes [68,69,70].

Astrocytes play a role in a variety of complex and essential functions in the healthy CNS, such as the maintenance of water and ion homeostasis and blood–brain barrier (BBB) integrity, as well as participation in tripartite synapses, all of which make astrocytes active actors in synaptic context [60,71]. Furthermore, astrocytes can inhibit or enhance overall levels of neuronal activity by releasing neurotransmitters. For many years astrocytes were classified into just two types, but now, according to their structure and anatomic location, up to four major classes of GFAP+ astrocytes are known to occur in the human brain: interlaminar astrocytes are located in layers I and II of the cortex; protoplasmic astrocytes reside in layers III and IV; astrocytes in varicose projections in layers V and VI; and fibrous astrocytes in white matter [72].

In the functional regulation of the microenvironment, astrocytes and oligodendrocytes release EVs, in order to facilitate the cell–cell communication and the activity of target cells [73,74,75].

The CNS macroglia cells are the ependymoglial cells derived from the neuroepithelium. They populate the interface between the brain parenchyma and the cavity of the ventricles in the CNS, and the central canal of the spinal cord. Macroglial cells appear with various shapes from cuboidal to columnar with cilia and microvilli on the apical surfaces to enhance absorbance and circulation of cerebral spinal fluid (CSF).

Ependymoglial cells are of three types: ependymocytes, which make contact with the basal lamina labyrinths (remnants of embryonic blood vessels) and with the ventricles where they contribute to the CSF movement [76,77]; choroid plexus epithelial cells, which secrete CSF; tanycytes, highly specialized ependymal cells that form a blood–CSF barrier and blood–CSF homeostasis [78]. Despite their role in CSF-barrier homeostasis regulation, there is not yet evidence for the exosomes’ secretion by the ependymal cells. However, they can be isolated by CSF [13,79], thus suggesting that they pass this barrier by still unknown mechanisms.

Differently from the previously described cells of the nervous system, the microglial cells originate from mesodermal hematopoietic cells that in mammals come from the yolk sac [80,81]. They serve as innate immunity elements of the CNS independently of blood cells. With self-renewal ability they act as unique tissue-resident macrophages involved in immune reactions and inflammatory diseases (Figure 1) [82].

To define the microglial cells just as macrophages would be an oversimplification because, in addition to their role in defending against bacterial and viral infections, they play a crucial role in the maturation of neural circuits by their “synaptic pruning” function [83]; they also produce brain-derived neurotrophic factor (BDNF) to survey mature neurons, mediate synapses, and remove myelin debris by phagocytosis [83,84].

Microglial cells have a great morphological plasticity and with their highly motile processes without moving their bodies constantly explore their environment. By screening the brain parenchyma these cells rapidly search for pathogens, signs of injury, or homeostatic disturbances [85,86]. Finally, the regulation of the neuronal plasticity by microglia may occur also by EVs releasing that have been reported implicated in the increase of neuronal synaptic activity in vitro and in vivo [75].

The functions of the nervous system and immune system are often considered independent from one another, however, this is a simplistic distinction, because in the regulation of the organism homeostasis, they are in constant communication [87,88]. This relationship was demonstrated long ago by the description of the association between peripheral neurons and mast cells, that are implicated in neuroinflammation [89]. The communication between neurons and mast-cells occurs through to several paracrine signals and also synapses, but the full understanding of this relation may open important scenarios pertaining to the onset of neuroinflammatory diseases [90].

In the PNS there are two types of neuroglia: Schwann cells (SCs) that myelinate axons; and satellite glial cells, that regulate nutrient and neurotransmitter levels in ganglia (Figure 1).

SCs are recognized as the PNS counterparts of the oligodendrocytes in CNS, as they are involved in the neuromuscular synapse formation and in wrapping myelin around neuronal axons to form the myelin sheath. This SC activity promotes the efficient and energy low-cost propagation of axon potentials via saltatory conduction by maintaining internodal—each myelin segment is flanked by unmyelinated nodes of Ranvier–myelin sheath thickness and length relative to the diameter of the corresponding axon [91]. SCs can perform many unique functions, including duplicating the roles of the astrocytes and microglia as seen in the central nervous system (CNS) [92]. The terminal Schwann cells (tSCs, also called non-myelinating SCs or perisynaptic SCs) have a role in “synapse elimination” during development and, throughout adult life [93], effect the regeneration of injured peripheral motor axons [94]. Schwann cells provide trophic support and transfer materials to damaged axons via exosomes [95]. Furthermore, they maintain developing synapses, and participate in synaptic pruning [96,97].

Satellite glial cells (SGCs) have the same origin as Schwann cells. Sensory ganglia of the dorsal roots of the spinal cord are composed of afferent neurons without a myelin sheath but lined by SGCs and connective tissue cells.

SGCs share many features with astrocytes, like the expression of glutamine synthetase and various neurotransmitter transporters. They cover axon terminals that make synaptic contacts on, or near, the neuronal somata, wrap around dendrites that emerge from neuronal somata to control the microenvironment and, similarly to astrocytes, influence synaptic transmission [98,99]. SGCs can be considered a substitute of the lacking BBB in sensory ganglia and have been shown to have phagocytic activity [98]. The role in microenvironment regulation and inflammation modulation by SGCs exosomes remains unknown [100,101].

### The Possible Two-Way Journey of Exosomes Released by CNS Cells

The roles of exosomes in the CNS may be as follows: on the one hand, they can be active components necessary for the development and protection of the CNS under physiological conditions [12,102,103], whereas on the other hand, they may participate in pathogenesis by favoring some neurodegenerative and neuroinflammatory phenomena, as suggested, for example, by the fact that microglial exosomes are found in high concentrations in patients with Alzheimer’s disease (AD) and exosomes produced by oligodendroglioma cells induce neuronal death [104].

**Figure 2:**
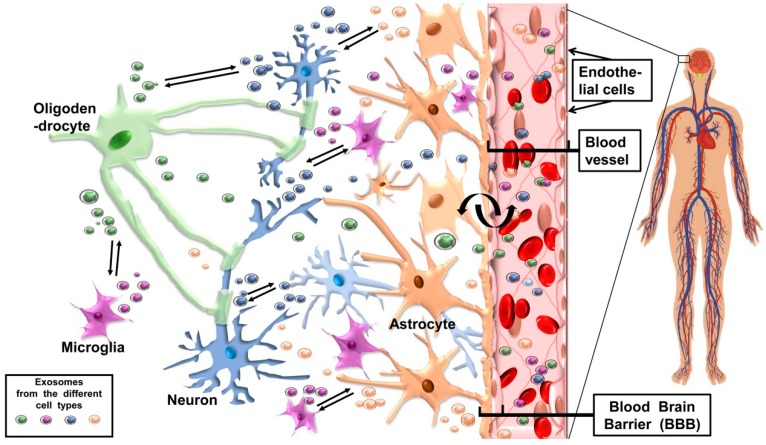
Schematic representation of the nervous tissue and exosome traffic. Some of the cells presented in Figure 1 are here seen in the central nervous tissue along with a blood vessel. Also present are epithelial cells lining the inside of the blood vessel, the blood–brain barrier (BBB) separating the lumen of the vessel from the nervous tissue, and exosomes secreted by the four types of nervous cells shown. Exosomes follow different routes, as indicated by double parallel arrows, from one cell to another or through the BBB they gain the general circulation and reach distant targets. Conversely, exosomes can traverse the BBB from inside the vessel into the nervous tissue and reach any of the nervous cell types in it.

Since exosomes are involved both in healthy and pathogenic state of CNS, it is not surprising that these vesicles are released by most of the CNS cells, including neurons, microglia, oligodendrocytes, astrocytes, and neural embryonic stem cells, Figure 2 [105,106]. Questions of great interest are whether these exosomes have the ability to cross the BBB and hematoliquor barrier; and whether they come from the CNS to the periphery, or they come from the periphery to the SNC in normal physiological and in pathological conditions.

Cells from malignant gliomas, i.e., primary tumors that arise from neuroglial stem or progenitor cells, produce and release in circulation exosomes with potential to induce malignant transformation of normal cells [107]. It has been reported that with a immunomagnetic exosome-RNA (iMER) analysis platform, it is possible to enrich glioblastoma (GBM)-derived exosomes from blood of patients, and compare the exosomes’ GBM-derived mRNA profiles against those of their cells of origin [108].

In amyotrophic lateral sclerosis (ALS), frontotemporal dementia (FTD), FTD-ALS, tauopathies, and Parkinson’s (PD) and Alzheimer’s (AD) diseases, exosomes migrate via blood and CSF carrying misfolded proteins or pro-inflammatory molecules [109,110,111].

Modified rabies virus glycoprotein (RVG)-targeted EVs were used to transport siRNA across BBB [112], that can specifically inhibit target genes in the brain [112]. Modified blood-borne macrophages were used to carry antioxidant proteins called nanozymes [113]. It was demonstrated that the therapeutic protein crossed the BBB and it was suggested that one of the mechanisms used by macrophages to transfer nanozymes to target recipient cells was the release of exosomes [113].

Furthermore, it was demonstrated that the uptake of EVs by neurons in vitro (neuronal rat adrenal pheochromocytoma cell line, PC12 cells) and in vivo neurons and microglial cells of mouse, is more efficient than that of other traditional carriers, i.e., liposomes [114].

## 3. Exosome-Mediated Cross-Talk between Cells in Neurogenesis and Neurohomeostasis

The development and maintenance of neuronal circuits in the CNS requires a complex series of events involving coordinated short- and long-distance communication between numerous cell types. Neurons interact continuously with each other and with glial cells through electrical signals and through chemical mediators. In chemical synapses, more common than electrical synapses in the human CNS, the transmission of signals is carried out by chemical mediators, named neurotransmitters. The neurotransmitters are secreted by the pre-synaptic cells inside vesicles that reach the post-synaptic cells, where multiple downstream events, both electric and molecular, are triggered by binding of the neurotransmitter to specific receptors [115]. In view of these phenomena, it is not surprising that neuronal cells may also release different types of EVs, such as exosomes that could have an impact on synaptic activity, in neurogenesis, and in the overall regulation of neurological activities. In this section, we will briefly describe known physiological functions of exosomes in CNS (Figure 2).

CNS neurons secrete exosomes to control the complex and coordinated communication among them and, with astrocytes and microglia, thus exosomes mediate a generalized cross-talk, in order to regulate neuronal regeneration and synaptic functions in development and adult life [116,117].

To the best of our knowledge, the first report regarding exosomes produced by neural cells is relatively recent. Glial cell lines overexpressing a prion protein (PrPsc) released exosomes as a way to spread the PrPsc and, these exosomes bearing PrPsc were infectious, contributing to the spread of prions throughout different areas of CNS and the whole organism [118]. Successively, it has been shown that neurons may exploit the exosome pathway to maintain homeostasis and regulate cell–cell interactions, for instance, as a way to discard unwanted proteins or degraded products. This hypothesis has been proposed to explain how primary cortical neurones in culture release exosomes in a controlled manner, while their composition is regulated by cell depolarisation [12]. The exosomes released are captured by neighbouring cells and the exosomal cargoes elicit distinct downstream events [12]. Other studies on exosome secretion from neurons have been conducted with embryonic neurons in culture. It was hypothesized that exosome release is a key mechanism during neurogenesis that seems to be necessary for protein removal, and is a consequence of the fusion of late endosomes with lysosomes, during the neurite elongation [119,120,121]. In addition, exosomes released from neurons may contribute to the local elimination of receptors at synapses undergoing plastic changes and escaping from the vesicles retrograde transport through the axon [59,122]. During neuronal remodelling, exosomes released by neurons could have a role in synapse elimination, stimulating microglial phagocytosis [123]. Furthermore, as the cytoplasmic calcium levels increase, MVBs’ fusion to the plasma membrane occurs and is followed the secretion of exosomes. This seems to be a mechanism used by neurons to detect the strength of the excitatory synapses and adjust them, a mechanism that might be necessary to regulate the functioning of synapses and maintain homeostasis during neuronal plastic changes [124]. In order to regulate extracellular glutamate levels and modulate synaptic activation, neurons communicate with astrocytes by secreting exosomes, which contain several regulatory molecules that are internalized by astrocytes, thus eliciting a neuronal-dependent modification of the expression of glutamate transporters (e.g., GLT1) [125]. Multiple interactions between glia-derived exosomes and neurons (Figure 2), also suggest a role of these vesicles in neural circuit development and maintenance, by promoting neurite outgrowth from hippocampal neurons and increased survival of cortical neurons [74].

Microglia-derived exosomes can modulate neuronal activity also via enhanced sphingolipid metabolism [75]. Inflammatory microglia-derived exosomes transfer their miRNA cargo (miR-146a-5p) to neurons determining the loss of excitatory synapses, suggesting a role during brain inflammation, probably silencing key synaptic genes [126].

In CNS, oligodendrocyte progenitor cells secrete exosome-like vesicles carrying myelin proteolipid protein (PLP), 2’3’-cyclic-nucleotide-phosphodiesterase (CNP), myelin basic protein (MBP), and myelin oligodendrocyte glycoprotein (MOG) [127]. The oligodendrocyte-derived exosomes may contribute to balanced production of myelin proteins and lipids and, therefore, these exosomes may be part of a mechanism of formation and control of myelin membrane biogenesis [103,127]. In adult CNS, during cell renewal and tissue regeneration, oligodendrocytes use the exosomal pathway to induce the microglia toward degradation of oligodendroglial membrane by macropinocytosis, without immune system activation [128]. The concomitant transfer of antigens from oligodendrocytes to microglia could be implicated in the pathogenesis of autoimmune conditions of the CNS. 

The fact that exosomes can reach the circulation and the CSF makes these vesicles likely means of long-distance communication and transport for bioactive molecules to be delivered to selected targets. Because of their capability to cross the BBB [129,130], and because their content reflects faithfully that of the cell of origin, circulating exosomes can reveal the status of the tissue from which they come and, thereby, provide an accurate means for early, minimally invasive (peripheral blood drawing) diagnosis of neurological diseases [22,41,131] (Figure 2).

On the other side of the matter, peripheral organs can influence the functions of CNS through exosomes [132,133]. The gut–brain axis is an example of an unconventional system of communication between the intestinal mucosa and brain, different from peripheral nerves.

The intestinal microbiota-derived EVs (named outer membrane vesicles, OMVs) can also enter the systemic circulation and pass through the BBB, inducing neuroinflammation that could be implicated in the pathogenesis of depressive disorders [134] and affect the BBB permeability [135]. The modality by which exosomes cross the BBB still remains unclear; however, this characteristic makes exosomes good candidates as biomarkers for diagnostics purposes, and for delivering therapeutic agents to neural tissues.

## 4. Role of Exosomes in Nervous System Pathogenesis and Theranostics

Progress in the medical sciences has been steady over the last few decades, encompassing the discovery of etiological agents, elucidation of pathogenic mechanisms, and development of new diagnostic techniques and therapeutic strategies. One of the major obstacles to improving patient management has been the heterogeneity of any given disease, which varies from patient to patient. Thus, personalized medicine has emerged to develop means of diagnosis and treatment for the management of each patient in accordance with its specific characteristics. Theranostics is one advance in this direction that also aims at combining diagnostic and therapeutic capabilities in a single agent [136]. Examples of theranostics agents are nanoparticles such as liposomes, polymers, micelles, solid (lipid) nanoparticles, antibodies, and now also exosomes, that can be modified and improved with drugs and imaging agents [137]. Nanoparticles have the ability to interact in a site-specific manner with biomolecules present on the cell membrane surface or inside the cell, co-delivering therapeutic and diagnostic/monitoring agents at the same time into diseased tissue. Appropriate targeting can be implemented using diverse strategies; for instance, to identify a cancer biomarker aberrantly expressed on the cell surface [138]. Theranostics has manifold advantages: (1) it can be carried out before, after, or during treatment; (2) the specific localization of the theranostics agents on a defined target reduces, or may even eliminate, possible side effects and can also help identify patients with susceptibility to side effects; (3) allows tumor homing: the nanometric size of the particles and the typical irregularity of blood vessels with dilated fenestrations, allow the extravasation and accumulation of nanoparticles into the tumor mass, improving the enhanced-permeability-and-retention (EPR) effect; and (4) it allows the achievement of a more effective individualized therapy for various diseases [139,140]. Theranostics is viewed as a significant step forward in non-invasive or minimally invasive treatment modalities with potential to accelerate drug development. However, theranostics has limitations in what pertains, for example, to the limited quantities of the therapeutic agent that can be delivered to the site where it is needed, the possibility of inducing immune reactions against the agent, manufacturing difficulties during nanoparticle production, and the need of elimination of toxic metabolites that might be generated during production and/or administration. In this regard, the biocompatibility, biodegradability, and toxicity of the materials used to prepare the theranostics agents and the pharmacokinetic and pharmacodynamic parameters of the compounds used have to be carefully evaluated before clinical use. In summary, the balance between benefits and disadvantages in each case must be critically assessed.

As described earlier, exosomes derived from different nervous system cells contain specific molecules or cell markers, e.g., oligodendrocyte-derived exosomes contain proteins of the myelin sheath; neuronal exosomes contain cell-adhesion proteins and receptor subunits; microglial-derived exosomes carry peptidases and cytokines. This suggests that exosomes have the ability to regulate and maintain functional cell homeostasis during health and under disease conditions. But on the other hand, exosomes can favor the disease mechanism rather than stop it, when they carry and deliver pathogenic molecules from the cell of their origin. This type of pathogenic role of exosomes has been observed in neuronal disorders with misfolded proteins (neurodegenerative, autoimmune, neuroinflammatory conditions), as will be discussed later.

### 4.1. Overview of CNS Disorders

**Figure 3:**
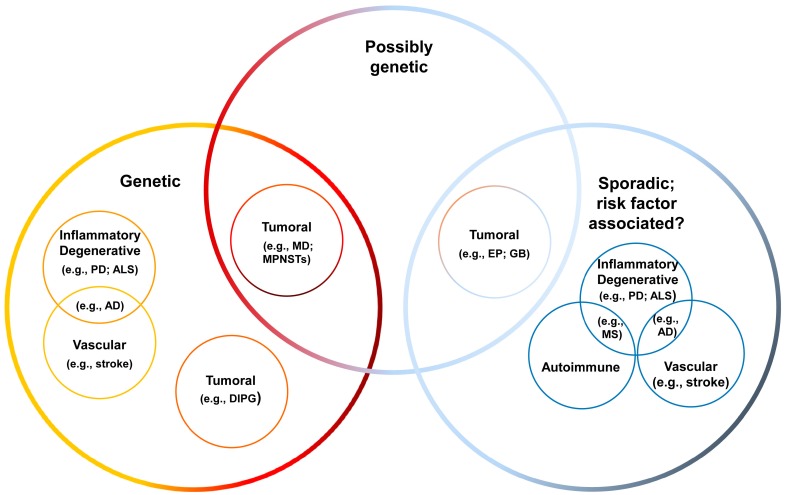
Diagrammatic representation of the various groups encompassing the neurological diseases presented in Table 1. It can be seen that according to their main etiopathogenic feature, neurological diseases can be classified into distinct groups. However, there are various examples in which a disease can be classified into more than one group because the etiopathogenic features are mixed, or incompletely understood. Abbreviations: AD, Alzheimer’s disease; ALS, amyotrophic lateral sclerosis; DIPG, diffuse intrinsic pontine gliomas; EP, ependymoma; GB, glioblastoma; MB, medulloblastoma; MPNSTs, malignant peripheral nerve sheath tumors; MS, multiple sclerosis; PD, Parkinson’s disease.

Neurologic disorders are numerous and diverse and can be caused by a variety of etiologic agents with many of the disorders being the consequence of the convergence of more than one etiopathogenic factor. An important group of neurological disorders are inherited, i.e., a mutated gene, or group of mutated genes are present in the genome of an individual which transmits it to its descendants. Some of these mutations are now well characterized [141,142,143]. However, the pathogenetic mechanisms of many of these genetic diseases are still poorly understood. Other disorders are caused by sporadic random gene mutations and are not heritable. Genetic polymorphisms; old age; gender; poor education, endocrine, immune and metabolic conditions; oxidative stress; inflammation; stroke; hypertension; diabetes; smoking; head trauma; depression; infection; tumors; vitamin deficiencies; and exposure to certain chemicals are considered risk factors that may contribute to the development of neurological diseases, including AD, PD and ALS, in individuals that are probably genetically pre-disposed [144]. Some gene mutations, random or inherited, affect development and functioning of the nervous system, leading to neuropathies, myopathies, epilepsies, ataxias, and degenerative disorders of the brain and spinal cord (Table 1, Figure 3) [145]. In what pertains to the etiology of nervous system tumors, there are still doubts and obscure situations: although genes associated with pathogenesis have been identified, other risk factors and comorbidities seem to also play determinant roles [146]. This situation is reflected in the classification of neurological disorders, which are encompassed in various large groups and subgroups, as summarized in Table 1 and in Figure 3. Because of the multifactorial mode of etiology, many neurological disorders can be assigned to more than one group or subgroup. Also, neurological disorders can be classified considering the location of the characteristic anatomic pathology, symptoms and signs, outcome, and other parameters.

The nervous system cells, Figure 1, can be the target of adaptive cellular and humoral immune responses, causing autoimmunity-induced damage [147]. Autoimmunity disorders involving the nervous system have been extensively investigated over the last few decades as in the case of multiple sclerosis (MS), which is characterized by inflammation with anti-myelin specific antibodies causing demyelination and neurodegeneration [148].

Neuroinflammatory disorders often include cases also classified within other groups. Neuroinflammation occurs as a direct response of the glial cells against injury, microbial infection, chemical substances, autoimmunity, or neurodegeneration of nervous tissue, but when the activation of microglial or macroglial cells becomes aberrant it can trigger acute inflammatory responses that can progress toward chronicity and have serious pathogenic consequences. Chronic inflammation is typically associated with some neurodegenerative diseases such as AD and PD. These and other disorders, for instance MS and ALS, differ in pathophysiology and can cause memory and cognitive impairments or affect a person’s ability to move, speak, and breathe. The outcome of a neurodegeneration is the loss of structural and functional neuronal integrity. Since there are several types of neurons and glial cells (Figure 1), their impairment causes a range of different symptoms and signs.

Neurovascular diseases, owing to defects of blood vessels supplying blood to CNS, can increase the risk of stroke. These neurovascular deficits are involved in pathogenic mechanisms in various neurodegenerative diseases, as for instance in AD [149].

Tumors are benign and malignant neoplasias of the CNS, PNS, autonomic nervous system, cranial nerves, and meninges (Table 1 and Figure 3). Genomic abnormalities can lead to glioblastoma, ependymomas, medulloblastomas, and diffuse intrinsic pontine gliomas. Malignant peripheral nerve sheath tumors (MPNSTs) are rare Schwann cell-derived neoplasms that can occur in individuals with autosomal dominant tumor susceptibility syndrome neurofibromatosis type 1 (NF1) [150].

### 4.2. Exosomes in Neurological Disorders

Studies on exosomes have contributed to increasing our current understanding of the pathogenesis of neurodegenerative disease. Since exosomal proteins were found accumulated in amyloid plaques in the brain of AD patients [151], the involvement of exosomes in AD pathogenesis deserves investigation. Secretion of exosomes may remove misfolded and/or aggregated proteins and transfer them to neighboring cells and, thereby, perpetuate the disease process. In vitro and in vivo experiments have confirmed that exosomes from neuronal cells contain precursors of amyloidogenic proteins and enzymes for the maturation of precursors [152,153,154,155]. The role of exosomes is not yet clear, but a possibility is that they could promote the spreading of beta-amyloid peptides and/or assist in the removal of neurotoxic beta-amyloid from cells [152,153].

The likelihood of exosome involvement in AD pathogenesis was also suggested by the finding of hyperphosphorylated tau protein in exosomes from neural tissue in culture and in human CSF [152]. Tau protein aberrantly accumulates in AD and, in this regard, microglial cells may participate in spreading tau protein through various brain regions by releasing exosomes as carriers [152,153]. Hyperphosphorylated tau protein in exosomes from transgenic mice would indicate that PTMs could enhance the abnormal process of tau formation and the spreading by exosomes [131,156]. In fact protein phosphorylation could be a signal required for their release by exosomes [157]. Exosomes may propagate tauopathies and, in AD, contribute to cognitive loss.

Currently, it is possible to diagnose AD when the disease is already established, e.g., when the patient has already developed dementia. The previous stages often remain asymptomatic, and these are the stages in which the patient could benefit most from treatment. Therefore, some sort of early diagnostic procedure is needed and, for this purpose, exosomes could be considered potentially useful biomarkers. Exosomes could act as Aβ scavengers binding Aβ to their surface and, subsequently, microglial cells would internalize “charged exosomes” and process them for degradation [158,159]. Consequently, exosomes derived from human adipose tissue-stem cells have been proposed for therapeutic degradation of Aβ plaques [160]. AD is associated also with chronic inflammatory responses. Microglia and astrocytes release inflammatory cytokines, and free radicals and oxidative stress molecules are present in the affected brain areas. As previously mentioned, Aβ is packaged into exosomes and the spreading from cell to cell and the promotion of amyloid plaque formation can initiate an inflammatory cascade [159]. Exosomes with the transactive response binding protein-43 (TDP43) are markers of amyotrophic lateral sclerosis and frontotemporal lobar degeneration [161]. Neuronal cells, but not astrocytes or microglia, release in vitro exosomes with the TDP43 full-length protein or its C-terminal fragments, both of which have been found in the brain of ALS patients [161,162]. Similarly to the transportation of protein tau in AD via exosomes with the propagation of tauopathy (discussed earlier), the release of TDP43 facilitates the progression of proteinopathy, neuroinflammation, and neurodegeneration [163]. In PD, alfa-synuclein aggregation is the pathological marker. This presynaptic neuronal protein has been shown to be secreted via exosomes and transferred to other normal cells [163,164], largely neurons and astrocytes, in which it had toxic effects causing death of the recipient cells [165,166].

Abnormalities in miRNA molecules are found in inflammatory cell populations or pathological samples of autoimmune disease [167]. It was demonstrated that exosomes carrying miRNAs can affect the recipient neural cells and dysregulate gene expression [165]. Almost 100 miRNAs have been found dysregulated in various affected tissue including brain, blood, and CSF of multiple sclerosis patients [168]. miRNA expression profiles in MS-derived exosomes compared to exosomes derived from healthy donors showed an overabundance of certain miRNAs, which were able to reduce the frequency of immune cells via inhibition of naïve-cell differentiation. Therefore, altered miRNA expression may play a role in pathogenesis of multiple sclerosis [168]. Exosome carrying miR-29b can affect neuronal function in HIV patients by suppressing the expression of the neuroprotective protein platelet-derived growth factor (PDGF)-B expression [169]. Also, in another infectious neurodegenerative disorder, prion disease, it has been demonstrated an alteration of exosomal miRNAs and, it has also been shown that prion protein scrapie (PrPSc) in neuronal exosomes can be passed to other cells via the exosomes and, thereby, infect neuronal and non-neuronal cells [118].

Tumor cells, derived from primary brain tumours or from metastases, use exosomes as packages to spread proteins and other molecules associated with malignancy [41,170]. Exosomes with their cargo would participate in the modulation of the tumor microenvironment, for instance by regulation of gene expression in the target cells and the functioning of the immune system, creating a pro-metastatic niche [171]. Tumor cell-derived exosomes can cross the BBB, which enhances tumor dissemination. This capability of tumor-derived exosomes to influence their environment has been demonstrated by showing that the exosomal microRNAs secreted by astrocytes target and inhibit the PTEN tumor suppressor gene expression in brain tumor cells, leading to enhanced oncogenicity [172]. Several findings confirmed the role of the brain tumor-derived exosomes in modulating immune functions by facilitating the induction of immunosuppressed phenotypes that favour the immune escape by means of their cargo of molecular mediators [158,159,173]. Moreover, glioblastoma-derived exosomes increase angiogenesis, which promotes tumor growth [174,175,176] and may support tumor dissemination also through the BBB [177]. Exosome-bearing tumorigenic mediators released by neuronal malignant cells have been isolated from serum of glioblastoma patients [174,178].

The current diagnostic approaches for most neurological disorders are limited to evaluation of clinical symptoms and radiologic signs. Consequently, the diagnosis can be tardive and treatment often produces negligible benefits. Therefore, it is necessary to find biomarkers that can be measured with minimally invasive procedures if progress in early diagnosis and reliable and timely assessment of response to treatment are to be achieved. Within this context, exosomes appear as suitable biomarker candidates as the key specimens of liquid biopsies. Efforts should be made to standardize assays with high specificity and sensitivity that would extract as much clinically relevant information as possible from exosomes. This approach is promising, considering that exosomes are a showcase of molecules present in their cells of origin.

### 4.3. Exosomes as Potential Therapeutic Tools

Some properties of exosomes make them, in principle, convenient for use as drug carriers for delivery to the CNS. For example, exosomes can cross physiological barriers and can interact with plasma-cell membranes, which may eventually lead to their penetration into target cells. Current knowledge suggests that exosomes may have advantages in comparison with other drug delivery agents such as liposomes, for example, in what concerns safety and selectivity, but more research is needed to determine their practical value in clinics. Some of these issues are discussed below.

In the last few years, research efforts have been focused on the manipulation of the exosomes’ content and their targeting to the CNS pathological sites for treating specific pathologies. The potential application of exosomes and EV in general, as therapeutic tools, has led to the development of new and advantageous therapies, particularly for brain tumors. Illustrative examples pertain to exosomes from bone marrow and mesenchymal stem cells (MSCs) [179] that were re-engineered to carry therapeutic drugs or other therapeutic molecules to diseased brain regions [180,181,182]. In one of the first studies, in a zebrafish model, endothelial cell-derived exosomes loaded with doxorubicin had the ability to pass through the BBB and reach brain tissue [180]. In other models, it has been found how the engineered exosomes enhanced the anti-tumor properties of immune cells [183] and could confer drug sensitivity [184,185].

In an animal experimental model of stroke, it has been shown that the intravenous administration of MSC-derived exosomes enhanced neurite remodelling, neurogenesis, and angiogenesis, leading to functional recovery [186]. The effect of neuronal damage recovery of MSC-derived exosomes was demonstrated also in a model of spinal cord injury, in which the beneficial effect was probably mediated by the transfer of miRNA-133b [187]. Mouse models have also been used in exosome-based therapies targeting AD. Exosomes were loaded by electroporation with exogenous siRNA and engineered to expose a brain-specific peptide and were delivered through the BBB [114]. This approach resulted in a significant and dose-dependent knockdown of the mRNA and protein for BACE1, a protease that produces N-terminal cleavage of amyloid precursor proteins that lead to Aβ aggregation [112].

In what pertains to the exosome-based strategies for the treatment of PD, the engineering of exosomes by electroporation with catalase can be mentioned [114]. In a mouse model of neuronal inflammation, intranasal administration of the engineered exosomes allowed them to interact with the target neighbouring neurons and deliver the antioxidant activity of catalase into these cells [114].

In a brain ischemia mouse model, engineered exosomes loaded with curcumin reached the target brain lesion after intravenous administration, supressing inflammation and apoptosis [188]. The efficiency of exosomes in passing through the BBB and in delivering a cargo protein was also demonstrated in another in vivo model [189]. Exosomes from naïve macrophages interacted with endothelial cells of microvessels in the BBB via native surface receptors.

The possible toxicity of exosomal preparations and the side effects of their administration in neuronal tissue are still to be explored further and various technical hurdles need to be overcome. However, exosomes have a great potential to be part of a versatile strategy to treat neurological disorders for all the reasons discussed above, such as the requirement of minimally invasive techniques, low immunogenicity, and ability to cross the BBB and reach the target pathological cells.

## 5. Conclusions

Incidence of CNS disorders is increasing worldwide, but no parallel progress in prevention and treatment occurs [190]. Novel treatment strategies based on the use of exosomes may help correct this deficiency and improve patient management. The reasons for the growing interest in exosomes as theranostics tools for CNS disorders, can be attributed to their characteristics and may be listed as follows: (1) the possibility of using exosomes as biomarkers, thus providing information about the status of the CNS; (2) exosomes are able to transverse the BBB; (3) they can be collected and administered with minimally invasive methods (e.g., peripheral blood and/or intranasal delivery); (4) their content can be manipulated as needed; and (5) their membrane proteins allows their targeting to precisely defined cell types, improving by engineering the specificity of any given treatment and, thus, reducing the side effects.

Despite the range of available information about exosomes as potential disease biomarkers and the increasing number of clinical trials on exosome-based drug delivery strategies, in cancer, for example [191,192], comparatively little is known about exosomes in the CNS. Therefore, much remains to be done to standardize the use of exosomes as therapeutic tools in CNS diseases.

## Figures and Tables

**Table 1:** Major neurological diseases and their main etiopathogenic features.

Disease	Main Etiopathogenic Feature ^b^						
	Genetic	Autoim-Mune	Inflam-Matory	Degener-Ative	Vascular	Tumoral	PGV ^a^
Multiple Sclerosis		x	x	x			
Alzheimer’s	x		x	x	x		
Parkinson’s	x		x	x			
Amyotrophic Lateral Sclerosis	x		x	x			
Ependymoma						x	x
Medulloblastoma	x					x	x
Diffuse intrinsic pontine glioma	x					x	
Glioblastoma	x					x	x
Malignant peripheral nerve sheath tumor	x					x	x

^a^ PGV, possible genetic variants. ^b^ The symbol “x” in table cell indicates that the etiopathogenic feature mentioned at the top of the column is present in the corresponding disease mentioned in the left-most column.
